# Supplementary material for: Macrophage-fibroblast JAK/STAT dependent crosstalk promotes liver metastatic outgrowth in pancreatic cancer
Source: Nat Commun. 2024 Apr 27;15:3593. doi: 10.1038/s41467-024-47949-3 (PMC11055860; doi:10.1038/s41467-024-47949-3)
Supplement: Supplementary file 1 — Supplementary Information [file 41467_2024_47949_MOESM1_ESM.pdf]

SUPPLEMENTARY INFORMATION

Title:

Macrophage-fibroblast JAK/STAT dependent crosstalk promotes liver metastatic outgrowth in pancreatic cancer

Authors:

Meirion Raymant[1], Yuliana Astuti[1], Laura Alvaro-Espinosa[2], Daniel Green[1], Valeria Quaranta[1], Gaia Bellomo[1], Mark Glenn[1], Vatshala Chandran-Gorner[1], Daniel H Palmer[1], Christopher Halloran[1], Paula Ghaneh[1], Neil C. Henderson[3,4], Jennifer P. Morton[5], Manuel Valiente[2], Ainhoa Mielgo[1], and Michael C. Schmid[1*]

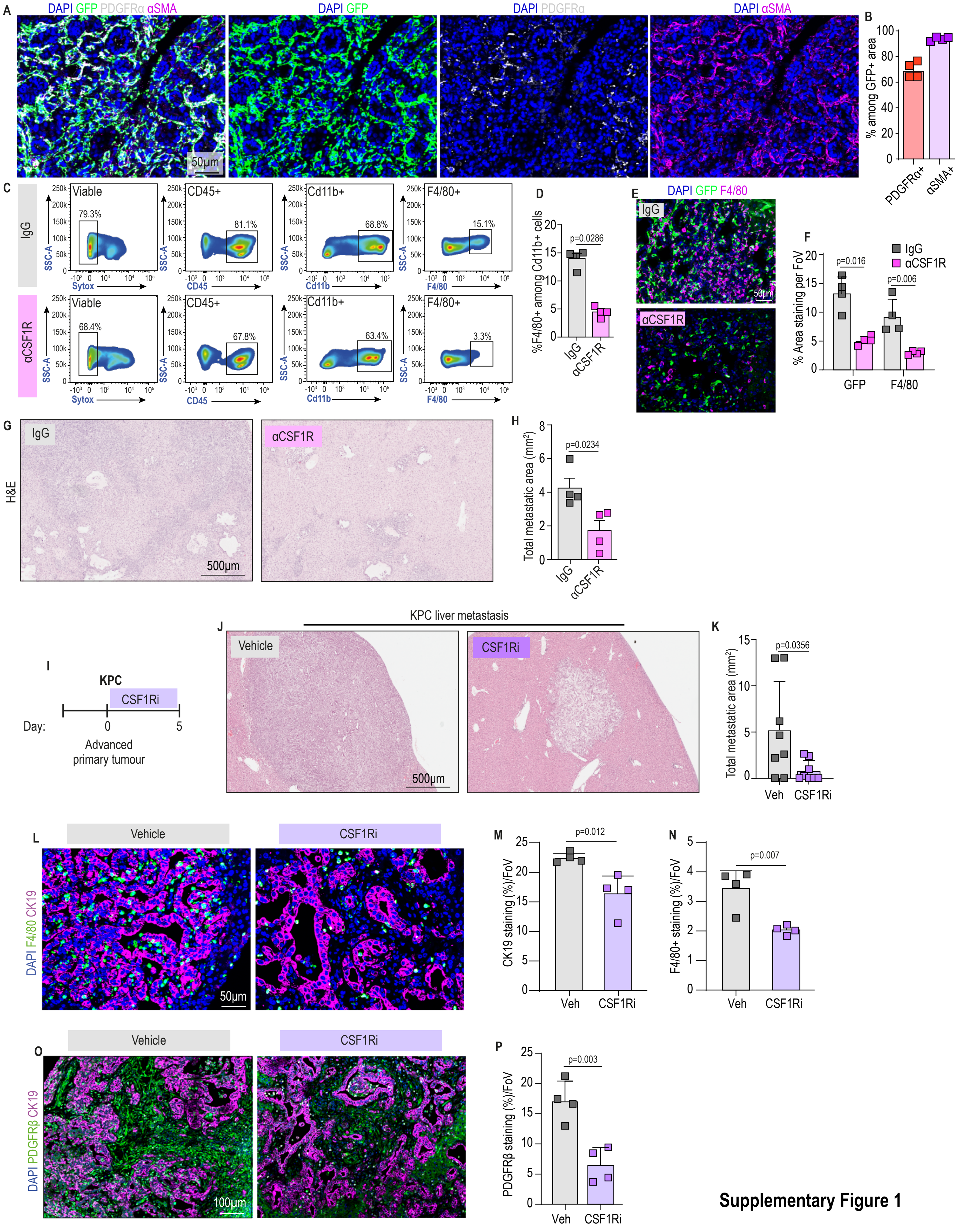

Supplementary Figure 1

Supplementary Figure 1 – Tumour associated macrophages promote metastatic outgrowth and mesenchymal expansion in preclinical models of experimental and spontaneous PDAC liver metastasis.

(A-B) Representative immunofluorescence image **(A)** and quantification **(B)** for the localisation of Pdgfrα and αSMA among GFP+ staining in metastatic tumours of *Pdgfrb*-GFP mice. Scale bar: 50µm. Percentage localisation is averaged. N=4 mice per group. Error bars, SD.

(C-D) Representative gating strategy **(C)** and quantification **(D)** for flow cytometric analysis of viable, $CD45^{+}$, $Cd11b^{+}$, $F4/80^{+}$ macrophages in metastatic tumours treated with IgG or αCSF1R therapy. Data is presented as averaged percentage abundance from n=4 mice per group. Error bars, SD. P value, two-tailed Mann-Whitney test.

(E-F) Representative immunofluorescence image **(E)** and quantification **(F)** of mesenchymal cells (GFP+) and macrophages (F4/80+) in metastatic tumours of *Pdgfrb*-GFP mice treated with IgG control or αCSF1R therapy. Scale bar: 50µm. Data is presented as averaged percentage staining. N=4 mice per group. Error bars, SD. P value, two-tailed unpaired t-test.

(G-H) Representative H&E staining **(G)** and quantification **(H)** of average metastatic area from *Pdgfrb*-GFP mice treated with IgG control or αCSF1R. Scale bar: 500µm. n=4 mice per group. Error bars, SD. P value, two-tailed unpaired t-test.

(I) Schematic of experiment design. KPC mice with advanced primary tumours were treated with CSF1Ri (AZD7505).

(J-K) Representative H&E staining **(J)** and quantification **(K)** of average metastatic area from KPC mice treated with CSF1Ri. Scale bar: 500µm. n=8 mice per group. Error bars, SD. P value, two-tailed unpaired t-test.

(L-N) Representative immunofluorescence image **(L)** and quantification of averaged percentage area occupied by **(M)** cancer cells (CK19+) and **(N)** macrophages (F4/80+) in liver metastasis of KPC mice treated with CSF1Ri. Scale bar: 50µm. n=4 mice per group. Error bars, SD. P value, two-tailed unpaired t-test.

(O-P) Representative immunofluorescence image **(O)** and quantification of averaged percentage area stained by **(P)** mesenchymal cells (PDGFRβ+) in liver metastasis (CK19+) of KPC mice treated with CSF1Ri. Scale bar: 100µm. N=4 mice per group. Error bars, SD. P value, two-tailed unpaired t-test. Source data are provided as a Source Data file.

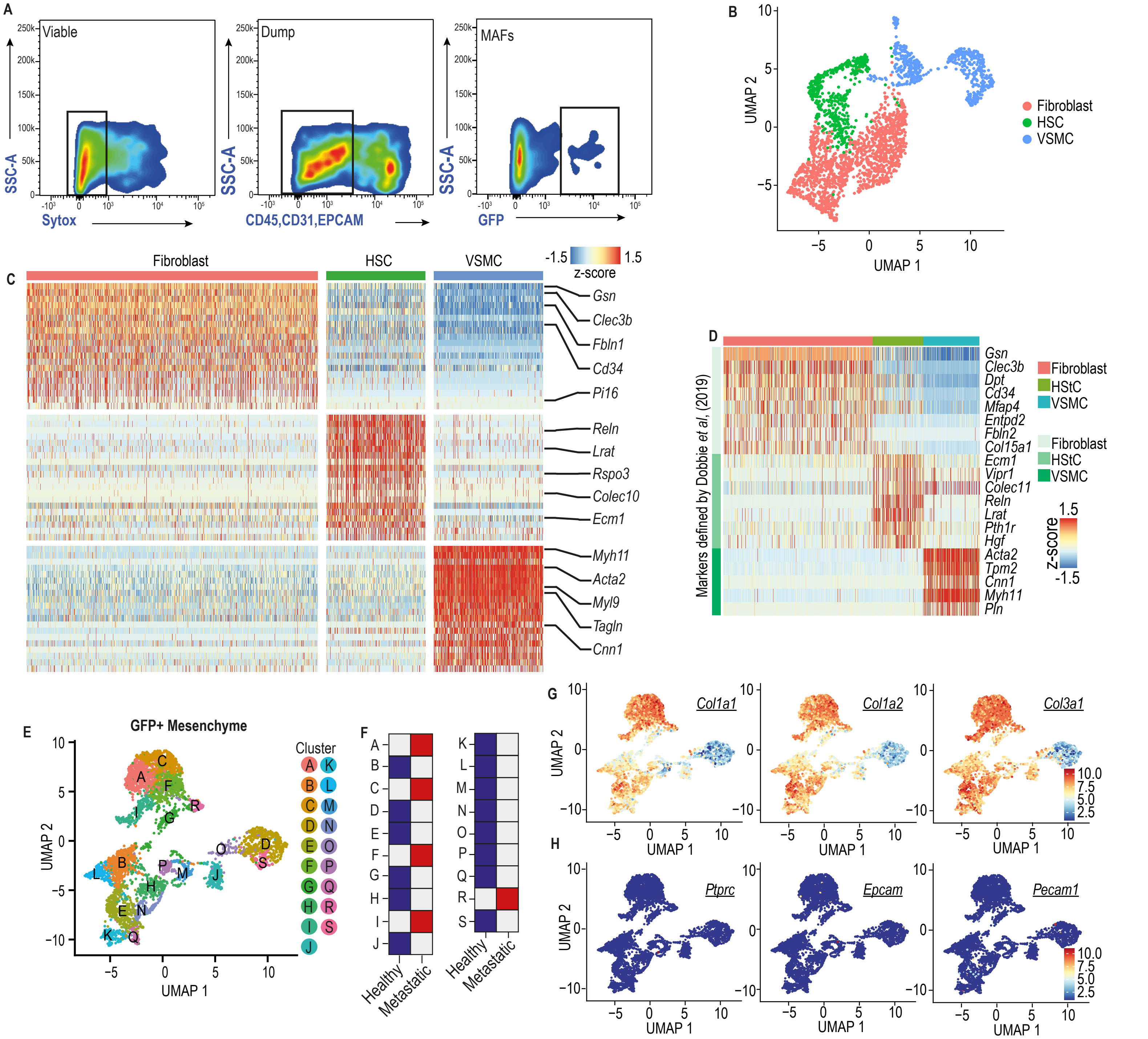

Supplementary Figure 2

Supplementary Figure 2 – Transcriptomic analysis of mesenchymal *Pdgfrb*-GFP+ cell populations identified by single cell RNA sequencing.

(A) Representative gating strategy for enrichment of GFP^+ cell fractions, for scRNAseq, by flow cytometric cell sorting of viable, $CD45^-$ (immune exclusion), $Epcam^-$ (epithelial exclusion), $CD31^-$ (endothelial exclusion), GFP^+ MAFs.

(B) UMAP plot of GFP+ cells isolated from healthy livers coloured by cluster membership depicting Fibroblasts (red), HStCs (green), and VSMCs (blue).

(C) Relative expression of indicated discriminative marker genes between Fibroblast, HStC and VSMC clusters from the UMAP in (**B**). Cells are displayed as columns and genes as rows. Colour scheme represents z-score distribution from -1.5 (blue) to 1.5 (red).

(D) Relative expression of indicated marker genes for Fibroblasts, HStCs, and VSMC signatures defined by Dobie *et al* [1] in comparison to clusters defined from the UMAP in (**B**). Cells are displayed as columns and genes as rows. Colour scheme represents z-score distribution from -1.5 (blue) to 1.5 (red).

(E) UMAP embedding of GFP+ mesenchyme from healthy and metastasis bearing livers separated by cluster membership.

(F) Classification of clusters from **(E)** into "Healthy" or "Metastatic". Metastatic = a cluster unique to metastasis-bearing dataset with <1% of cells originating from the Healthy dataset. Blue = Healthy, Red = Metastatic.

(G) UMAP plots depicting expression of common fibroblast activation markers (*Col1a1*, *Col1a2*, and *Col3a1*) in GFP+ mesenchyme of healthy and metastasis bearing liver. Colour indicates marker gene expression (blue = low; red = high).

(H) UMAP plots depicting expression levels of common markers of immune cells (*Ptprc*), epithelial cells (*Epcam*) and endothelial cells (*Pecam1*) in GFP+ mesenchyme of healthy and metastasis-bearing livers. Colour indicates marker gene expression (blue = low; red = high).

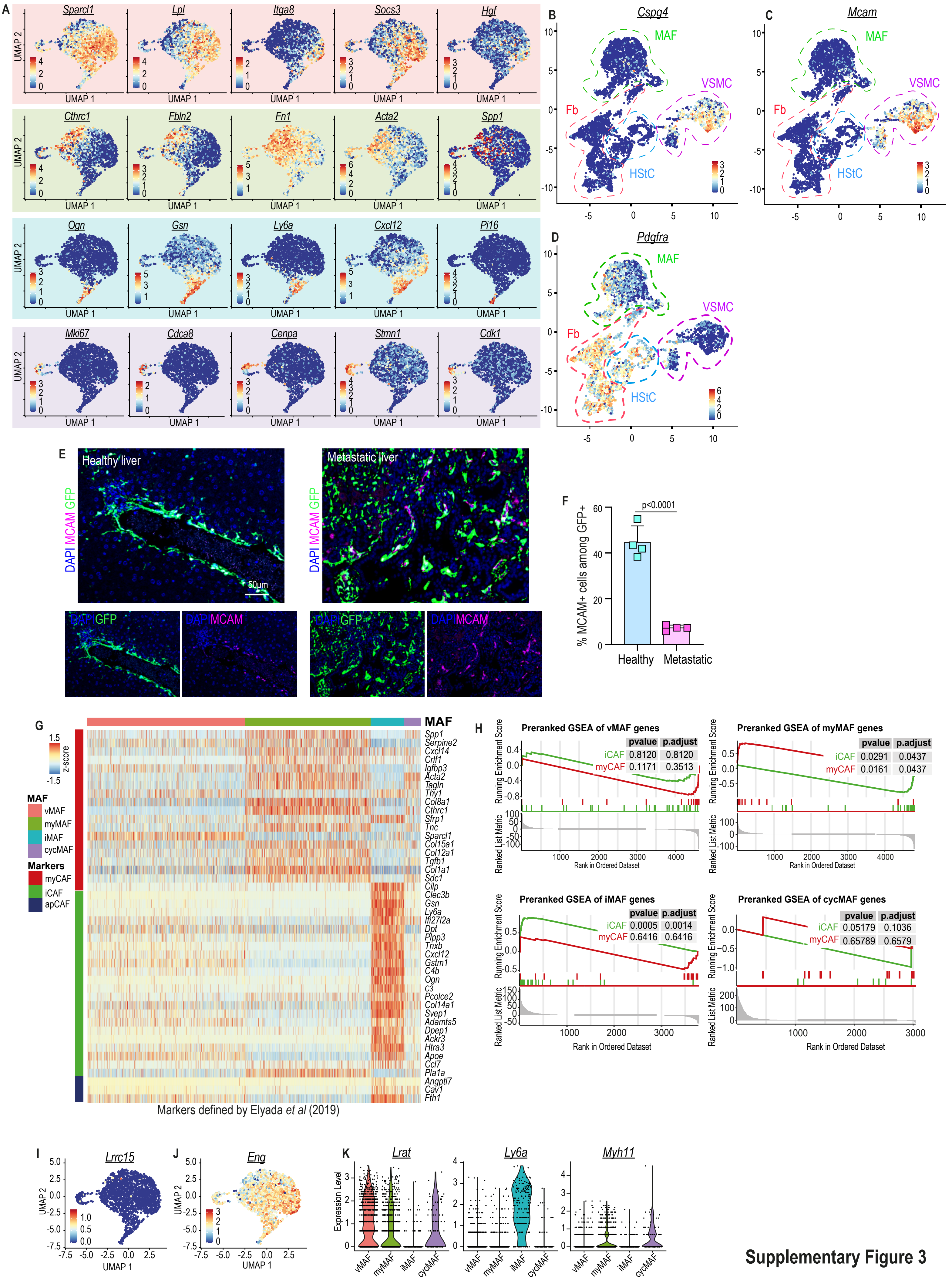

Supplementary Figure 3

Supplementary Figure 3 – Transcriptomic analysis of GFP+ MAF subpopulations identified by single cell RNA sequencing

(A) UMAP plots depicting expression levels of the top markers associated with vMAF, myMAF, iMAF, and cycMAF populations. Colour indicates marker gene expression (blue = low; red = high). Background shading of UMAP plots corresponds to cluster identity.

(B-D) UMAP plots depicting expression levels of **(B)** *Cspg4*, **(C)** *Mcam*, and **(D)** *Pdgfra* across healthy and metastatic mesenchymal cells. Cell identity is defined on the plot. Colour indicates marker gene expression (blue = low; red = high).

(E-F) Representative immunofluorescence image **(E)** and quantification **(F)** of averaged percentage of MCAM+GFP+ double positive cells, among GFP+ cells, in healthy and metastatic livers of *Pdgfrb*-GFP mice. Scale bar: 50µm. N=4 mice per group. Error bars, SD. P value, two-tailed unpaired t-test.

(G) Enrichment of indicated marker genes for myCAF, iCAF, and apCAFs, defined by Elyada *et al* [2] among vMAF, myMAF, iMAF, and cycMAF clusters. Cells are displayed as columns and genes as rows. Colour scheme represents z-score distribution from -1.5 (blue) to 1.5 (red).

(H) Gene set enrichment analysis (GSEA) of vMAF, myMAF, iMAF, and cycMAF populations against signatures of myCAF and iCAF phenotypes defined by Elyada *et al* [2].

(I) UMAP plot depicting the expression of *Lrrc15* in GFP+ MAFs, Colour indicates marker gene expression (blue = low; red = high).

(J) UMAP plot depicting the expression of *Eng* in GFP+ MAFs, Colour indicates marker gene expression (blue = low; red = high).

(K) Violin plot depicting expression of *Lrat*, *Ly6a,* and *Myh11* expression, markers of HStC, Fb, and VSMCs, respectively, among vMAFs, myMAFs, iMAFs and cycMAFs. Source data are provided as a Source Data file.

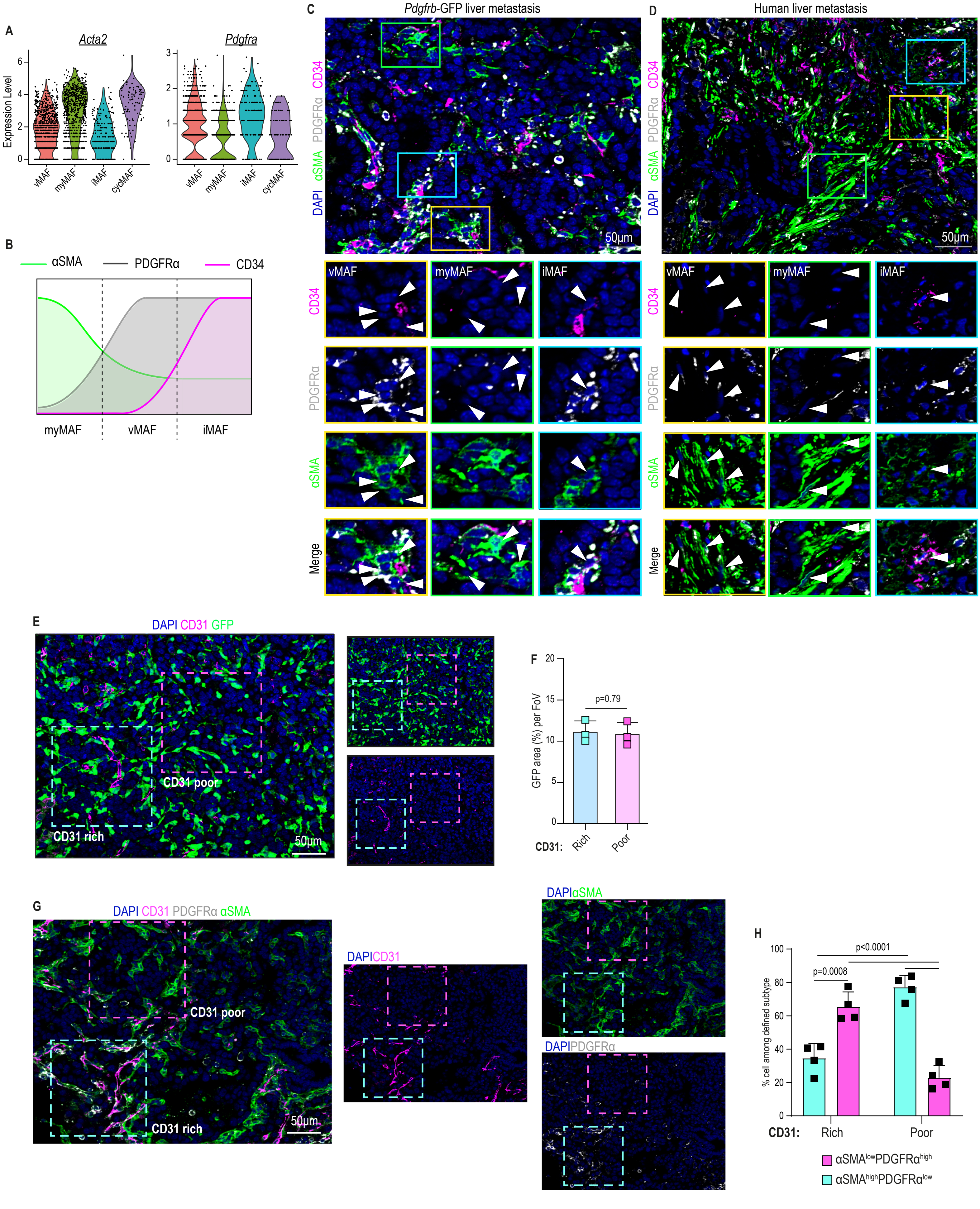

Supplementary Figure 4

Supplementary Figure 4 – Cross-species and spatial annotation of MAF subtypes in metastatic PDAC identified by single cell RNA sequencing.

(A) Violin plot of *Acta2* and *Pdgfra* expression across vMAF, myMAF, iMAF, and cycMAF populations.

(B) Illustration depicting the gradated expression of αSMA, PDGFRα and CD34, corresponding to the transcriptional expression across MAF subtypes. Dotted line indicates the interphase of each cluster.

(C-D) Representative immunofluorescence image of αSMA, PDGFRα, and CD34 in **(C)** experimental liver metastases in *Pdgfrb*-GFP mice (n=3 mice), and **(D)** biopsies of metastatic PDAC patients (n=3 patients). Regions of interest highlight MAF subtypes as defined in **(B)**. Higher magnification images for the defined regions of interest are displayed in their corresponding colours. Yellow rectangle = vMAF (αSMA^{low}, PDGFRα^{high}, CD34^{low}; Green rectangle = myMAF (αSMA^{high}, PDGFRα^{low}, CD34^{low}; Blue rectangle = iMAF (αSMA^{low}, PDGFRα^{high}, CD34^{high}). White arrowheads highlight the defined MAF subtype. Scale bar: 50µm.

(E-F) Representative immunofluorescence image **(E)** and quantification **(F)** of the distribution of MAFs (GFP+) in regions defined rich (blue dashed rectangle) and poor (pink dashed rectangle) in vasculature (CD31+). Scale bar: 50µm. Data presented as averaged percentage area occupied by GFP+ staining. N=3 mice per group. Error bars, SD. *P* value, two-tailed unpaired *t*-test.

(G-H) Representative immunofluorescence image **(G)** and quantification **(H)** of the distribution of αSMA^{low}PDGFRα^{high} and αSMA^{high}PDGFRα^{low} cells across regions rich (blue dashed rectangle) and poor (pink dashed rectangle) in vasculature (CD31+). Scale bar: 50µm. Data is presented as averaged percentage distribution. N=4 mice per group. Error bars, SD. *P* value, two-way ANOVA with Tukey's multiple comparisons. Source data are provided as a Source Data file.

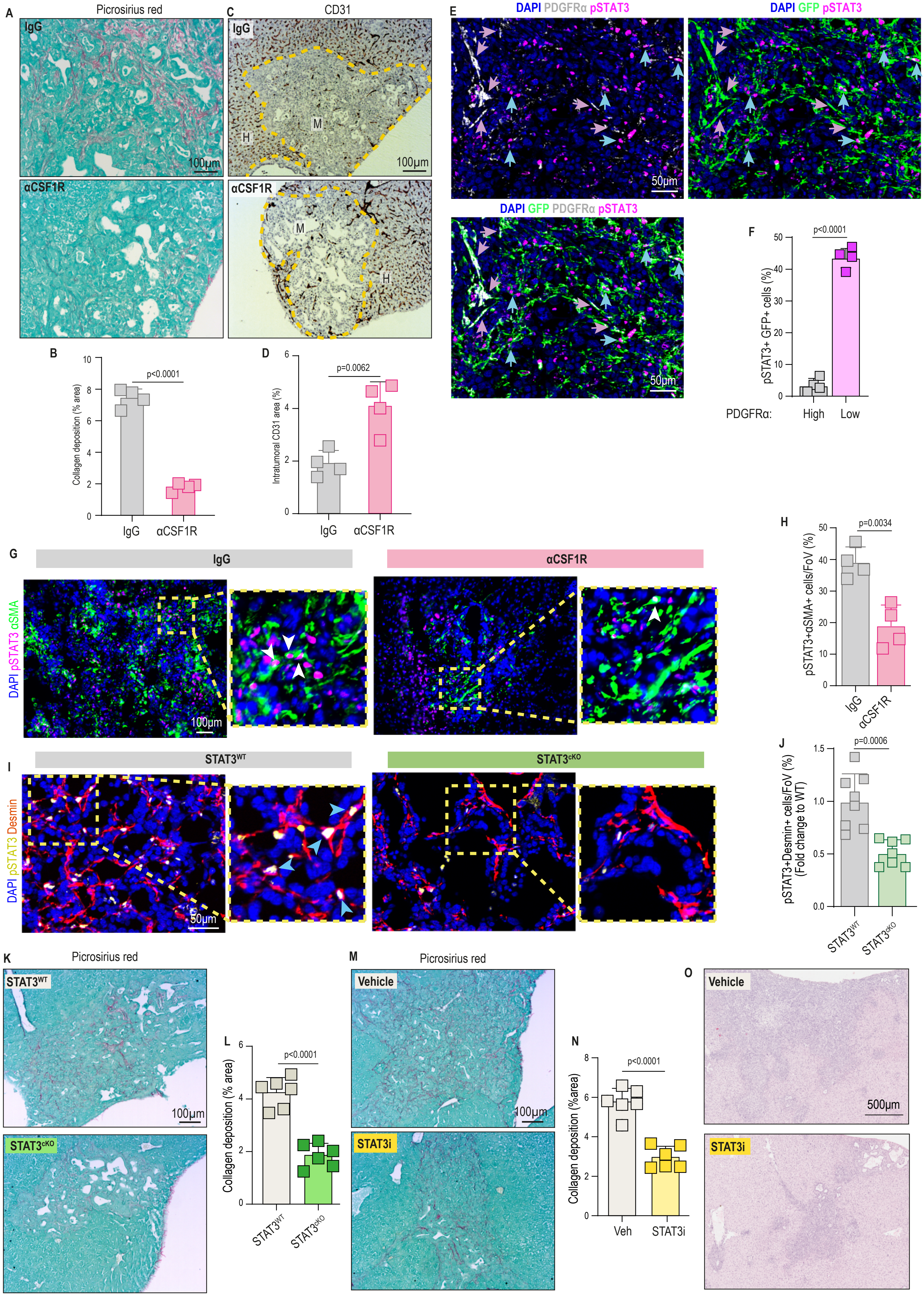

Supplementary Figure 5

Supplementary Figure 5 – Pharmacological and genetic inhibition of STAT3 in MAFs supresses collagen deposition and metastatic outgrowth.

(A-B) Representative image **(A)** and quantification **(B)** of collagen deposition in metastatic tumours of IgG and αCSF1R treated mice. Scale bar, 100 µm. Data presented as averaged percentage area stained. N=4 mice per group. Error bars, SD. *P* value, two-tailed unpaired *t*-test.

(C-D) Representative immunohistochemical image **(C)** and quantification **(D)** of intra-tumoral CD31 (DAB – brown), defined by dashed line, in metastatic tumours of IgG and αCSF1R treated mice. H = healthy; M = metastatic. Scale bar, 100 µm. Data presented as averaged percentage area stained. N=4 mice per group. Error bars, SD. *P* value, two-tailed unpaired *t*-test.

(E-F) Representative immunofluorescence images **(E)** and quantification **(F)** of pSTAT3+ PDGFRα^{high} and PDGFRα^{low} cells, among GFP+ cells, in metastatic tumours of *Pdgfrb*-GFP mice. Light blue arrowheads, PDGFRα^{low}GFP+pSTAT3+ cells. Pink arrowheads: PDGFRα^{high} GFP+pSTAT3+ cells. Scale bar, 50 µm. Data presented as averaged percentage of pSTAT3+ cells. N=4 mice per group. Error bars, SD. *P* value, two-tailed unpaired *t*-test.

(G-H) Representative immunofluorescence images **(G)** and quantification **(H)** of JAK/STAT active (pSTAT3+) myMAFs (αSMA+) in metastatic tumours of *Pdgfrb*-GFP mice treated with IgG, or αCSF1R. Arrowheads indicate pSTAT3+αSMA+ cells. Scale bar, 100 µm. Data presented as averaged percentage double positive cells, among αSMA+ cells. N=4 mice per group. Error bars, SD. *P* value, two-tailed unpaired *t*-test.

(I-J) Representative immunofluorescence images **(I)** and quantification **(J)** of JAK/STAT active (pSTAT3+) HStCs (desmin+) in metastatic tumours of n=8 STAT3^{WT} and n=7 STAT3^{cKO} mice. Arrowheads indicate pSTAT3^{+} desmin^{+} cells. Scale bar, 50 µm. Data presented as averaged percentage of double positive cells, among desmin+ cells, relative to control group. Scale bar, 100 µm. Error bars, SD. *P* value, two-tailed unpaired *t*-test.

(K-N) Representative image and quantification of collagen deposition in metastatic tumours derived from **(K-L)** STAT3^{WT} and STAT3^{cKO} mice, or **(M-N)** vehicle and STAT3i *Pdgfrb*-GFP mice. Scale bar: 100 µm. Data presented as averaged percentage area stained. N=6 mice per group. Error bars, SD. *P* value, two-tailed unpaired *t*-test.

(O) Representative H&E staining in metastatic *Pdgfrb*-GFP mice treated with STAT3i, or vehicle control. Scale bar: 500µm. N=6 mice per group. Source data are provided as a Source Data file.

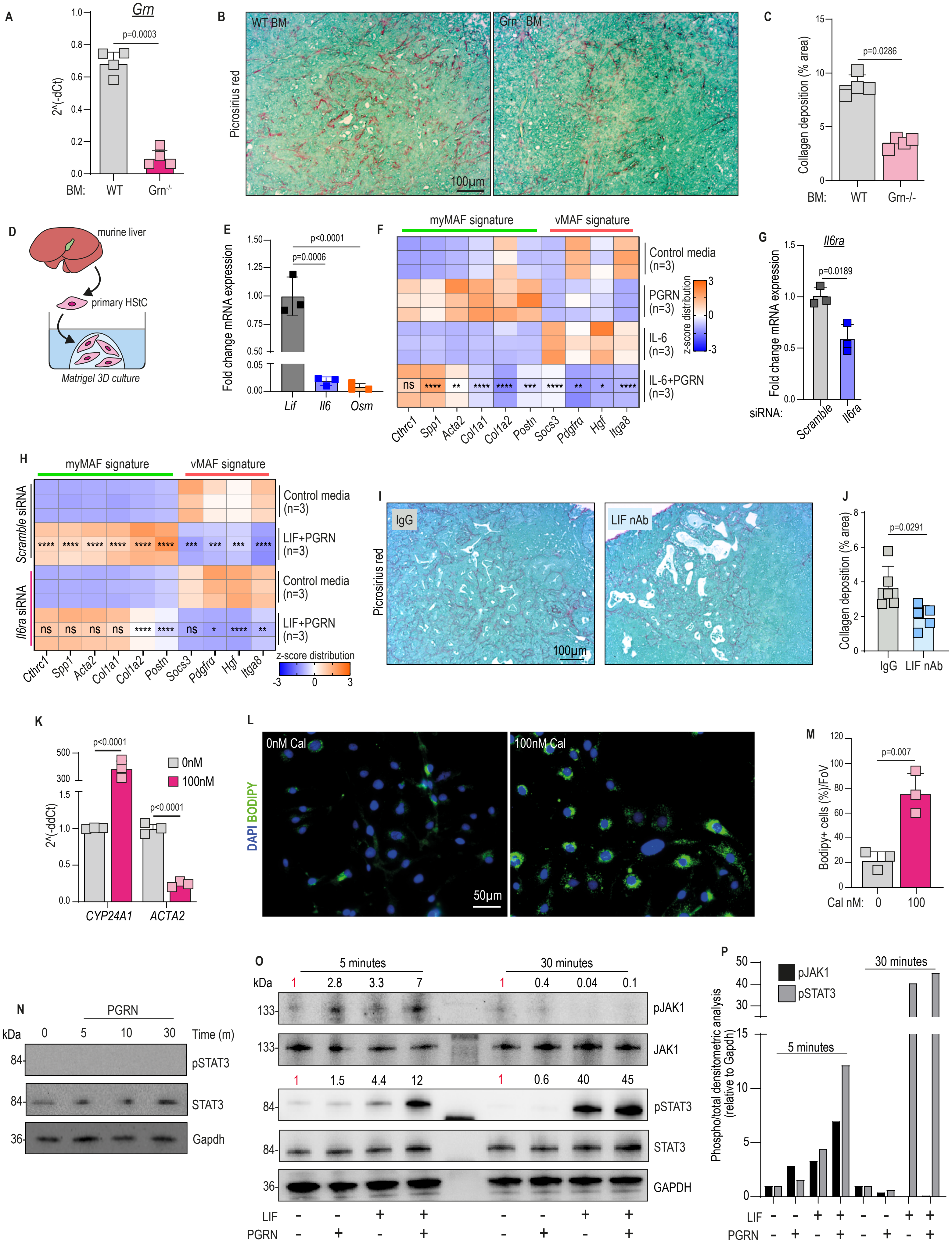

Supplementary Figure 6

Supplementary Figure 6 – Co-stimulation of LIF and Progranulin induces a JAK/STAT active myMAF phenotype *in vitro*.

(A) qPCR of *Grn* mRNA levels. n=4 mice averaged per group. Error bars, SD. *P* value, two-tailed unpaired *t*-test.

(B-C) Representative image **(B)** and averaged percentage area **(C)** of collagen deposition. Scale bar: 100 µm. N=4 mice per group. Error bars, SD. *P* value, two-tailed Mann-Whitney test.

(D) Illustration depicting isolation of primary murine HStCs 3D-matrigel culturing.

(E) qPCR of *Lif*, *Il6*, and *Osm* mRNA levels in KPC-cancer cells. averaged across n=3 independent experiments. Error bars, SD. *P* value, one-way ANOVA and Tukey's multiple comparisons.

(F) qPCR of vMAF and myMAF genes in primary HStCs. Data is presented as heatmap of z-scores. N=3 independent experiments, represented by rows. *P*-value, one-way ANOVA with Tukey's multiple comparisons. Significance shown: progranulin+IL6 vs progranulin.

(G) qPCR of *Il6ra* mRNA levels in primary HStCs treated with *Scramble* or *Il6ra* siRNA. N=3 independent experiments. Error bars, SD. *P* value, two-tailed unpaired *t*-test.

(H) qPCR of vMAF and myMAF genes in primary HStCs. Data is presented as heatmap of z-scores. N=3 independent experiments. *P* value, one-way ANOVA with Tukey's multiple comparisons. Significance shown: *Scramble*-LIF+PGRN vs *Scramble*-control media; and *Il6ra*-LIF+progranulin vs *Scramble*-LIF+PGRN.

(I-J) Representative image **(I)** and averaged percentage area **(J)** of collagen deposition. Scale bar: 100 µm. N=5 mice per group. Error bars, SD. *P* value, two-tailed unpaired *t*-test.

(K) qPCR of *CYP24A1* and *ACTA2* relative mRNA levels in LX2 cells stimulated with 100 nM of calcipotriol (Cal). N=3 biological replicates are averaged. Error bars, SD. *P* value, two-tailed unpaired *t*-test.

(L-M) Representative immunofluorescence images **(L)** and averaged percentage of BODIPY+ LX2 cells **(M)**. Scale bar: 50 µm. N=3 independent experiments. Error bars, SD. *P* value, two-tailed unpaired *t*-test.

(N) Immunoblot of pSTAT3 in LX2 cells stimulated with progranulin over 30 minutes. Gapdh, loading control. Experiment was repeated three time with similar results.

(O-P) Immunoblot **(O)** and densitometric analysis **(P)** of pJAK/JAK and pSTAT3/STAT3 in LX2 cells stimulated with Progranulin, LIF, or both, for 5 and 30 minutes. Loading control, Gapdh. Experiment was repeated three times with similar results. Source data and exact p values are provided as a Source Data file.

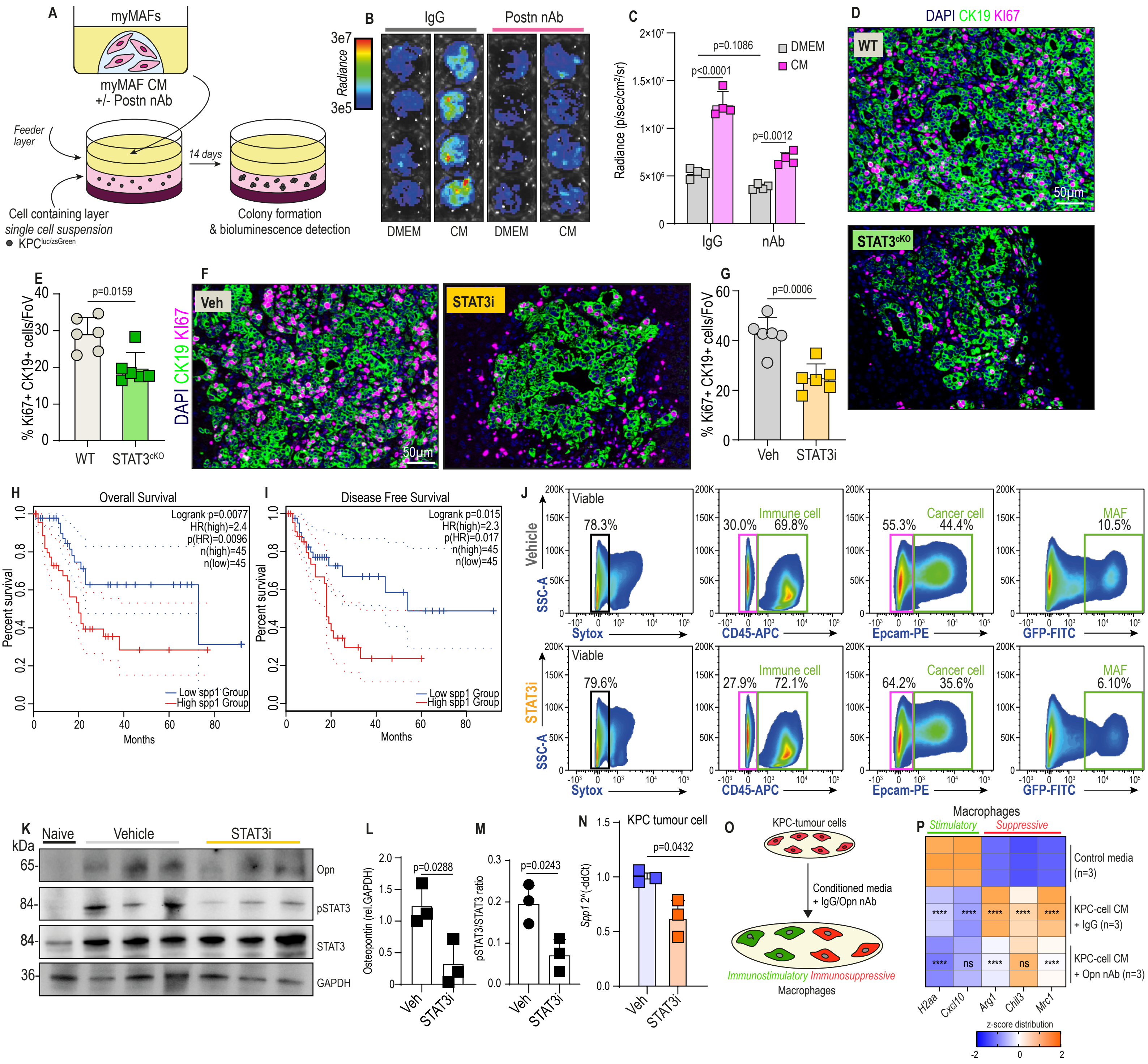

Supplementary Figure 7

Supplementary Figure 7 – myMAF-derived periostin promotes anchorage independent outgrowth of KPC cells.

(A) Schematic of KPC cell colony formation assay.

(B-C) Representative images **(B)** and averaged bioluminescence **(C)** from KPC-colonies exposed to myMAF CM, in the presence or absence of periostin nAb. N=4 independent experiments. Error bars, SD. *P* value, two-way ANOVA with Tukey's multiple comparisons.

(D-E) Representative immunofluorescence image **(D)** and quantification **(E)** of proliferating (KI67+) cancer cells (CK19+) in metastatic tumours of $STAT3^{WT}$ and $STAT3^{cKO}$ mice. Scale bar, 50 µm. Data is presented as averaged percentage of double positive cells, among CK19+ cells. N=6 mice per group. Error bars, SD. P value, two-tailed Mann-Whitney test.

(F-G) Representative immunofluorescence image **(F)** and quantification **(G)** of proliferating (KI67+) cancer cells (CK19+) in metastatic tumours of vehicle and STAT3i *Pdgfrb*-GFP mice. Scale bar: 50 µm. Data is presented as averaged percentage of double positive cells, among CK19+ cells. N=6 mice per group. Error bars, SD. P value, two-tailed unpaired t-test.

(H-I) Kaplan-Meier survival plot comparing percent **(H)** overall- and **(I)** disease free- survival of PDAC patients. Groups are divided by low (blue) or high (red) *SPP1* expression.

(J) Representative gating strategy for sorting immune (CD45+), cancer (Epcam+), and MAF (GFP+) cells from metastases of vehicle and STAT3i treated *Pdgfrb*-GFP mice.

(K-M) Immunoblotting **(K)** and densitometric analysis of **(L)** osteopontin and **(M)** pSTAT3/STAT3, relative to GAPDH, in bulk-tissue of healthy liver, and vehicle or STAT3i treated metastatic tumours. N=3 independent biological samples are averaged per group. Error bars, SD. *P* value, two-tailed unpaired *t*-test.

(N) qPCR of *Spp1* relative mRNA expression in KPC cancer cells treated with STAT3i, or vehicle control. Data averaged from n=3 independent experiments. Error bars, SD. *P* value, two-tailed unpaired *t*-test.

(O-P) Schematic of experimental design **(O)** and qPCR **(P)** of immunostimulatory and immunosuppressive genes in BMDMs exposed to KPC-cancer cell CM, pre-treated with either osteopontin nAb or IgG control. N = 3 independent experiments. *P* value, one-way ANOVA with Tukey's multiple comparisons. Significance: KPC-cell CM + IgG vs Control media; KPC-cell CM + OPN nAb vs KPC-cell CM + IgG. Source data and exact p values are provided as a Source Data file.

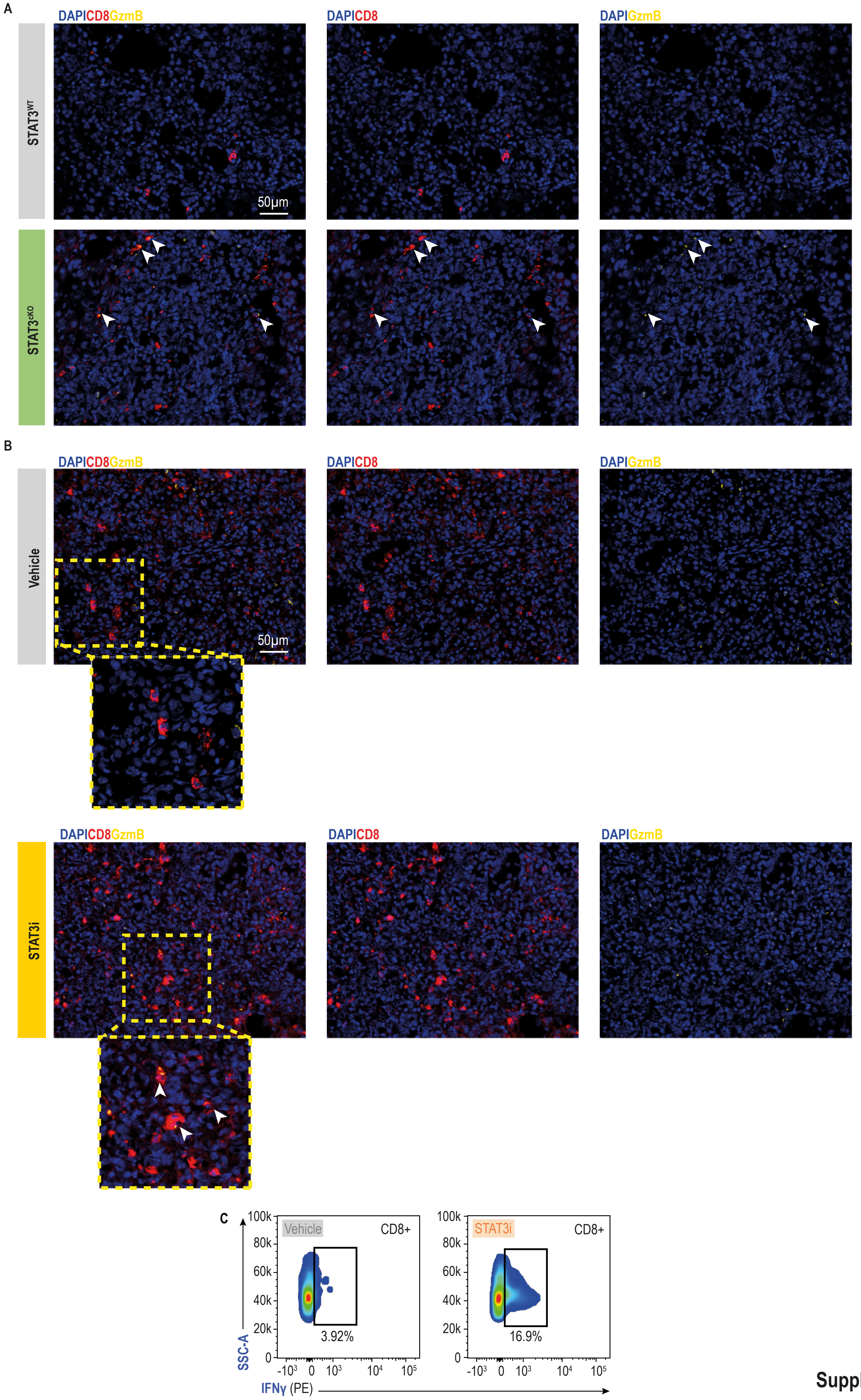

Supplementary Figure 8

Supplementary Figure 8 – Pharmacological and genetic STAT3i in myMAFs restores CD8+ T cell infiltration and cytotoxicity.

(A) Representative immunofluorescence images of cytotoxic (Gzmb+) T cells (CD8+) in metastatic tumours of $STAT3^{WT}$ and $STAT3^{cKO}$ mice. Arrowheads indicate GzmB+CD8+ double positive cells. Scale bar: 50 µm. n=6 mice per group.

(B) Representative immunofluorescence images of cytotoxic (GzmB+) T cells (CD8+) in metastatic tumours of Vehicle and STAT3i (Silibinin) treated mice. Arrowheads indicate GzmB+CD8+ double cells. Scale bar: 50 µm. n=6 mice per group.

(C) Representative gating strategy for flow cytometry analysis of $CD8^+$ T cell activation depicted by $IFN\gamma^+$ $CD8^+$ T cells isolated from metastatic tumours of *Pdgfrb*-GFP mice, treated with vehicle or STAT3i. n=4 mice per group.

Supplementary table 1. Name, product code, company, and dilution of all antibodies utilised in this study.

Antibody list for Immunostaining.				
**Name**	**Product ID/clone**	**Company**	**Dilution**	**Method**
Gapdh	G9545	Sigma Aldrich	1:10000	Immunoblotting
STAT3	9139	Cell signalling technology	1:1000	Immunoblotting
pSTAT3	9145	Cell signalling technology	1:1000	Immunoblotting
JAK1	3344	Cell signalling technology	1:1000	Immunoblotting
pJAK1	74219	Cell signalling technology	1:1000	Immunoblotting
Sortilin	Ab16640	Abcam	1:1000	Immunoblotting
alpha Tubulin	T6199	Sigma Aldrich	1:5000	Immunoblotting
Osteopontin	AF808	R&D Systems	1:1000	Immunoblotting
Anti-mouse IgG-HRP	7076	Cell signalling technology	1:5000	Immunoblotting
Anti-rabbit IgG-HRP	7074	Cell signalling technology	1:5000	Immunoblotting
Anti-goat IgG-HRP	ab97120	Abcam	1:20000	Immunoblotting
CD45	30F-11	Biolegend	1:50	Flow Cytometry
F4/80	BM8	Biolegend	1:50	Flow Cytometry
CD11b	M1/70	Biolegend	1:50	Flow Cytometry
CD31	390	Biolegend	1:50	Flow Cytometry
Epcam	G8.8	Biolegend	1:50	Flow Cytometry
CD8a	53-6.7	Biolegend	1:50	Flow Cytometry
IFNg	XMG1.2	Biolegend	1:50	Flow Cytometry
Ki67	ab15580	Abcam	1:1000	Immunohistochemistry
CD31	ab222783	Abcam	1:100	Immunohistochemistry
αSMA	ab17007	Abcam	1:100	Immunohistochemistry
pSTAT3	9145	Cell signalling technology	1:100	Immunohistochemistry
Cytokeratin 19	ab53119	Abcam	1:1000	Immunofluorescence
Cytokeratin 19	ab52625	Abcam	1:1000	Immunofluorescence
F4/80	70076	Cell signalling technology	1:100	Immunofluorescence
GFP	ab6556	Abcam	1:100	Immunofluorescence
PDGFRβ	ab69506	Abcam	1:100	Immunofluorescence
CD68	M081401-2	DAKO	1:200	Immunofluorescence
PDGFRα	ab203491	Abcam	1:100	Immunofluorescence
αSMA	ab5694	Abcam	1:50	Immunofluorescence
αSMA	ab7817	Abcam	1:50	Immunofluorescence
CD34	ab8158	Abcam	1:100	Immunofluorescence

CD34	M082329-2	DAKO	1:100	Immunofluorescence
pSTAT3	9145	Cell signalling technology	1:100	Immunofluorescence
CD31	77699	Cell signalling technology	1:100	Immunofluorescence
MCAM	Ab75769	Abcam	1:100	Immunofluorescence
Desmin	ab32362	Abcam	1:100	Immunofluorescence
Ki67	ab15580	Abcam	1:1000	Immunofluorescence
Ym-1	60130	Stem cell Technologies	1:50	Immunofluorescence
CD8a	ab22378	Abcam	1:50	Immunofluorescence
Granzyme B	AF165	R&D Systems	1:50	Immunofluorescence
Anti-mouse IgG Alexa Fluor(AF)-488	ab150105	Abcam	1:300	Immunofluorescence
anti-rabbit IgGAF-488	ab150077	Abcam	1:300	Immunofluorescence
anti-rat IgG AF-594	ab150160	Abcam	1:300	Immunofluorescence
anti-rabbit IgG AF-594	ab250080	Abcam	1:200	Immunofluorescence
anti-rat IgG AF-647	ab150155	Abcam	1:300	Immunofluorescence
anti-rabbit IgG AF488	406404	Biolegend	1:300	Immunofluorescence
anti-rabbit IgG AF594	406418	Biolegend	1:300	Immunofluorescence
anti-rabbit IgG AF647	406414	Biolegend	1:300	Immunofluorescence
Alexa Fluor 488 tyramide reagent	B40953	ThermoFisher	1:500	Immunofluorescence
Alexa Fluor 594 tyramide reagent	B40957	ThermoFisher	1:500	Immunofluorescence
Alexa Fluor 647 tyramide reagent	B40958	ThermoFisher	1:500	Immunofluorescence
Superboost Goat anti-rabbit poly HRP	B40962	ThermoFisher	As instructed per manual	Immunofluorescence
Strepavidin tyramide superboost kit	B49035	ThermoFisher	As instructed per manual	Immunofluorescence
Goat anti-rat biotinylated antibody	B4-9400-1.5	2B scientific	1:500	Immunofluorescence
Goat anti-rabbit biotinylated antibody	BA-1000-1.5	2B scientific	1:500	Immunofluorescence

Supplementary table 2. List of all primers used in this study for quantitative real time-PCR.

Primer list for quantitative real time-PCR					
**Species**	**Gene symbol**	**Assay name**	**Sequence (5'-3')**	**Cat. No.**	**Company**
Murine	*Gapdh*	Mm_Gapdh_3_SG		QT01658692	Qiagen
Murine	*Lif*	Mm_Lif_1_SG		QT00111090	Qiagen
Murine	*Acta2*	Mm_Acta2_1_SG		QT00140119	Qiagen
Murine	*Postn*	Mm_Postn_1_SG		QT00150759	Qiagen
Murine	*Col1a1*	Mm_Col1a1_1_SG		QT00162204	Qiagen
Murine	*Col1a2*	Mm_Col1a2_1_SG		QT01055572	Qiagen
Murine	*Pdgfra*	Mm_Pdgfra_1_SG		QT00140021	Qiagen
Murine	*Tnc*	Mm_Tnc_1_SG		QT00106176	Qiagen
Murine	*H2aa*	Mm_H2-Aa_1_SG		QT01061858	Qiagen
Murine	*Cxcl10*	Mm_Cxcl10_1_SG		QT00093436	Qiagen
Murine	*Arg1*	Mm_Arg1_1_SG		QT00134288	Qiagen
Murine	*Chi3l3*	Mm_Chi3l3_1_SG		QT00108829	Qiagen
Murine	*Mrc1*	Mm_Mrc1_1_SG		QT00103012	Qiagen
Murine	*Spp1*	Mm_Spp1_1_SG		QT00157724	Qiagen
Murine	*Il6*	Mm_Il6_1_SG		QT00098875	Qiagen
Murine	*Il6ra*	Mm_Il6ra_1_SG		QT00098168	Qiagen
Murine	*Osm*	MM_Osm_1_SG		QT00263193	Qiagen
Murine	*Cthrc1*		Forward: GGACC TCTTCCCA TCGAAGC Reverse: TCCCTTCACAGA GTCCTTCCA	Custom	Sigma Aldrich
Murine	*Hgf*		Forward: GCTCCT CCCTTCCC TACTCG Reverse: CCCACATCATGC TTGCAGTT	Custom	Sigma Aldrich
Murine	*Lpl*		Forward: GGAGAA GCCATC CGTGTGAT Reverse: CTCAGGCAGAGC CCTTTCTC	Custom	Sigma Aldrich
Murine	*Itga8*		Forward: ACACGTTC CTCAAG AGAAAGAA Reverse: TCGGAGTGGCCCA AATAACC	Custom	Sigma Aldrich
Murine	*Socs3*		Forward: GGGAGC CCCTTTG TAGACTT Reverse: CATCCCGGGGA GCTAGT	Custom	Sigma Aldrich
Human	*GAPDH*	Hs_GAPDH_SG		QT00079247	Qiagen
Human	*LIF*	Hs_LIF_1_SG		QT00001442	Qiagen

Human	*ACTA2*	Hs_ACTA2_1_SG		QT00088102	Qiagen
Human	*CYP24A1*	Hs_CYP24A1_1_SG		QT00015428	Qiagen

Supplementary References

1. Dobie, R. *et al.* Single-Cell Transcriptomics Uncovers Zonation of Function in the Mesenchyme during Liver Fibrosis. *Cell Rep* **29**, 1832-1847 e1838 (2019).
2. Elyada, E. *et al.* Cross-Species Single-Cell Analysis of Pancreatic Ductal Adenocarcinoma Reveals Antigen-Presenting Cancer-Associated Fibroblasts. *Cancer Discov* **9**, 1102-1123 (2019).
