# Supplementary material for: Macrophage-fibroblast JAK/STAT dependent crosstalk promotes liver metastatic outgrowth in pancreatic cancer
Source: Nat Commun. 2024 Apr 27;15:3593. doi: 10.1038/s41467-024-47949-3 (PMC11055860; doi:10.1038/s41467-024-47949-3)
Supplement: Supplementary file 3 — Description of Additional Supplementary Files [file 41467_2024_47949_MOESM3_ESM.pdf]

Description of Additional Supplementary Files

Supplementary Data 1: Differentially expressed genes (DEGs) among the defined GFP+ MAF subpopulations.

Top DEGs between vMAF, myMAF, iMAF, and cycMAF clusters detected using the Wilcoxon rank-sum test as implemented in the FindMarkers function. After Bonferroni correction, genes with an adjusted P value less than 0.05 were deemed significant.
