# Supplementary material for: Macrophage-fibroblast JAK/STAT dependent crosstalk promotes liver metastatic outgrowth in pancreatic cancer
Source: Nat Commun. 2024 Apr 27;15:3593. doi: 10.1038/s41467-024-47949-3 (PMC11055860; doi:10.1038/s41467-024-47949-3)
Supplement: Supplementary file 5 — Reporting Summary [file 41467_2024_47949_MOESM5_ESM.pdf]

nature portfolio

Corresponding author(s): Michael C Schmid

Last updated by author(s): Mar 28, 2024

Reporting Summary

Nature Portfolio wishes to improve the reproducibility of the work that we publish. This form provides structure for consistency and transparency in reporting. For further information on Nature Portfolio policies, see our Editorial Policies and the Editorial Policy Checklist.

Statistics

For all statistical analyses, confirm that the following items are present in the figure legend, table legend, main text, or Methods section.

n/a	Confirmed	
☐	☒	The exact sample size (*n*) for each experimental group/condition, given as a discrete number and unit of measurement
☐	☒	A statement on whether measurements were taken from distinct samples or whether the same sample was measured repeatedly
☐	☒	The statistical test(s) used AND whether they are one- or two-sided<br>*Only common tests should be described solely by name; describe more complex techniques in the Methods section.*
☒	☐	A description of all covariates tested
☐	☒	A description of any assumptions or corrections, such as tests of normality and adjustment for multiple comparisons
☐	☒	A full description of the statistical parameters including central tendency (e.g. means) or other basic estimates (e.g. regression coefficient) AND variation (e.g. standard deviation) or associated estimates of uncertainty (e.g. confidence intervals)
☐	☒	For null hypothesis testing, the test statistic (e.g. *F*, *t*, *r*) with confidence intervals, effect sizes, degrees of freedom and *P* value noted<br>*Give P values as exact values whenever suitable.*
☒	☐	For Bayesian analysis, information on the choice of priors and Markov chain Monte Carlo settings
☒	☐	For hierarchical and complex designs, identification of the appropriate level for tests and full reporting of outcomes
☒	☐	Estimates of effect sizes (e.g. Cohen's *d*, Pearson's *r*), indicating how they were calculated

Our web collection on statistics for biologists contains articles on many of the points above.

Software and code

Policy information about availability of computer code

Data collection	scRNAseq was performed on Illumina NovaSeq 6000<br>RT qPCR was run on AriaMx Real Time PCR system<br>Flow cytometry was run on FACSCanto II<br>Cell sorting was performed on FACSAria III<br>Fluorescence imaging of cells was performed on Zeiss LSM 800 microscope<br>Brightfield or fluorescence imaging of tissues was performed on Zeiss Axio Observer Z1 microscope<br>Imaging of western blot membrane was performed on Chemidoc system
Data analysis	Sequencing data were processed through CellRanger 3.0.2 and analysed in R 4.0.2 using the Seurat library. All code used to analyse data in this study is available on GitHub at https://github.com/CBFLivUni/Raymant-et-al-2023<br>Gene ontology enrichment analysis was performed using g:Profiler & REVIGO<br>Gene set enrichment analysis was performed in R using the clusterProfiler package<br>qPCR data were analysed using AriaMx software<br>Flow cytometry data were analysed using FlowJo v10<br>Microscopy imaging data were analysed using FIJI and Zeiss Zen software<br>Dnesitometric analysis was performed using FIJI and BioRad ImageLab Software v6.1<br>Statistical tests were performed using GraphPad Prism 8

For manuscripts utilizing custom algorithms or software that are central to the research but not yet described in published literature, software must be made available to editors and reviewers. We strongly encourage code deposition in a community repository (e.g. GitHub). See the Nature Portfolio guidelines for submitting code & software for further information.

Data

Policy information about availability of data

All manuscripts must include a data availability statement. This statement should provide the following information, where applicable:

- Accession codes, unique identifiers, or web links for publicly available datasets
- A description of any restrictions on data availability
- For clinical datasets or third party data, please ensure that the statement adheres to our policy

The publicly available scRNAseq data of MAFs derived from metastasis-bearing Pdgfrb-GFP mice generated in this study has been deposited in the Gene Expression Omnibus (GEO) database under accession code GSE232335 [https://www.ncbi.nlm.nih.gov/geo/query/acc.cgi?acc=GSE232335]. The remaining data are available within the Article, Supplementary Information, and Source Data. Source data are provided with this paper as a Source Data file. Any further information is available from the corresponding author upon request.

Research involving human participants, their data, or biological material

Policy information about studies with human participants or human data. See also policy information about sex, gender (identity/presentation), and sexual orientation and race, ethnicity and racism.

Reporting on sex and gender	This information has not been collected
Reporting on race, ethnicity, or other socially relevant groupings	This information has not been collected
Population characteristics	Liver biopsy samples were collected from patients with treatment-naïve, advanced PDAC with pathologically confirmed liver metastasis. Patients were 53-64 years old, however age was not a consideration for sample collection.
Recruitment	Liver biopsies were part of diagnostic samples from consented patients with treatment-naïve, advanced PDAC at the Royal Liverpool University Hospital. No participation selection was applied in this study. All patients consenting to donating biological samples were included in this study. No biases were present.
Ethics oversight	This study complies with all relevant ethical regulations. Studies involving the use of liver metastasis biopsy and blood samples from patients with treatment-naive, advanced PDAC were accessed using the PINCER platform study, approved by the National Research Ethics Service Committee North West, Greater Manchester REC15/NW/0477. All individuals provided written informed consents on approved institutional protocol. All animal studies were conducted in accordance with UK Home Office regulations under project license P16F36770.

Note that full information on the approval of the study protocol must also be provided in the manuscript.

Field-specific reporting

Please select the one below that is the best fit for your research. If you are not sure, read the appropriate sections before making your selection.

☒ Life sciences ☐ Behavioural & social sciences ☐ Ecological, evolutionary & environmental sciences

For a reference copy of the document with all sections, see nature.com/documents/nr-reporting-summary-flat.pdf

Life sciences study design

All studies must disclose on these points even when the disclosure is negative.

Sample size	No statistical methods were used to pre-determine sample sizes but we used adequate numbers of samples that would provide statistically significant results, based on our previous experiences with the intrasplenic tumour models (PMID: 38355776; PMID: 35022267; PMID: 29789416). For animal studies, at least 3 mice were analysed from each group. For image analysis, at least 3 fields of view were averaged per biological replicate.
Data exclusions	No data were excluded from analysis
Replication	Biological replicates are defined for each experiment in the figure legends. In vitro experiments were repeated three times unless otherwise specified in the figure legends.
Randomization	In vivo experiments were not randomised, however mice with comparable age, body weight, and sex, were assigned into control and experimental groups. Sex was not considered in the study design, and in all studies sex was randomly assigned to both groups, with the exception of STAT3i survival study, in which only female mice of 6-8 weeks were utilised. For cell culture experiments, individual wells were randomly assigned to treatment groups.
Blinding	Investigators were not blinded to allocation during experiments and outcome assessments. Treatments, data collections, and analyses were mostly performed by the same person, so blinding was not possible. In some instances, in vivo dosing, terminations, and imaging data acquisition was acquired/supported by a different person who did not have knowledge of expected treatment outcome.

Reporting for specific materials, systems and methods

We require information from authors about some types of materials, experimental systems and methods used in many studies. Here, indicate whether each material, system or method listed is relevant to your study. If you are not sure if a list item applies to your research, read the appropriate section before selecting a response.

Materials & experimental systems

n/a	Involved in the study
☐	☒ Antibodies
☐	☒ Eukaryotic cell lines
☒	☐ Palaeontology and archaeology
☐	☒ Animals and other organisms
☒	☐ Clinical data
☒	☐ Dual use research of concern
☐	☐ Plants

Methods

n/a	Involved in the study
☒	☐ ChIP-seq
☐	☒ Flow cytometry
☒	☐ MRI-based neuroimaging

Antibodies

Antibodies used

Immunoblotting:
Gapdh (G9545, Sigma Aldrich, 1:10,000); STAT3 (9139, Cell Signaling Technology, 1:1000); pSTAT3 (9145, Cell Signaling technology, 1:1000); pJAK1 (74219, Cell Signaling Technology); JAK1 (3344, Cell Signaling Technology); Sortilin (Ab16640, Abcam, 1:1000); alpha tubulin (T6199, Sigma Aldrich; 1:5000), Osteopontin (AF808, R&D Systems, 1:1000), Anti mouse IgG HRP (7076, cell signaling technology, 1:5000), anti-rabbit IgG HRP (7074, Cell signaling technology, 1:5000), Anti-goat IgG HRP (ab97120, abcam, 1:20,000).

Flow cytometry:
CD45 (30F-11, Biolegend, 1:50); F4/80 (BM8, Biolegend, 1:50); Cd11b (M1/70. Biolegend, 1:50); CD31 (390, Biolegend, 1:50); Epcam (G8.8, Biolegend, 1:50); CD8a (53-6.7, Biolegend, 1:50); IFNg (XMG1.2, Biolegend, 1:50).

Immunohistochemistry:
Ki67 (ab15580, abcam, 1:1000); CD31 (ab222783, abcam, 1:100); aSMA (ab7817, Abcam, 1:100); pSTAT3 (9145, Cell signaling technology, 1:100).

Immunofluorescence:
Cytokeratin-19 (ab53119, abcam, 1:1000), Cytokeratin-19, ab52625, Abcam, 1:1000); f4/80 (70076, Cell signaling technology, 1:100); GFP (ab6556, abcam, 1:100); PDGFRb (ab69506, abcam, 1:100); CD68 (MO81401-2, dako, 1:200); Pdgfra (ab203491, abcam, 1:100); aSMA (ab5694, abcam, 1:50), aSMA (ab7817, abcam, 1:50); CD34 (ab8158, abcam, 1:100), CD34, (M082329-2, DAKO, 1:100); pSTAT3 (9145, cell signaling technology, 1:100), CD31 (77699, cell signaling technology, 1:100); MCAM (ab75769, abcam, 1:100); desmin (ab32362, abcam, 1:100); Ki67 (ab15580, abcam, 1:1000); YM-1 (60130, Stem cell technologies, 1:50); CD8a (ab22378, abcam, 1:50), GranzymeB (AF1865, R&D systems, 1:50). Anti-mouse IgG AF-488 (ab150105, abcam, 1:300), anti-rabbit IgG AF-488 (ab150077, abcam, 1:300); Anti-rat IgG AF 594 (ab150160, abcam, 1:300); Anti-rabbit IgG AF-594 (ab150080, abcam, 1:200); Anti-rat IgG AF-647 (ab150155, abcam, 1:300); Anti-rabbit IgG AF488 (406404, biolegend, 1:300); Anti-rabbit IgG AF594 (406418, Biolegend, 1:300); Anti-rabbit IgG AF647 (406414, Biolegend, 1:300); Alexa Fluor 488 Tyramide reagent (B40953, ThermoFisher Scientific); Alexa Fluor 594 Tyramide Reagent (B40957, ThermoFisher Scientific); Alexa Fluor 647 Tyramide reagent (B40958, ThermoFisher Scientific); Superboost Goat Anti-rabbit Poly HRP (B40962, ThermoFisher Scientific); Streptavidin Tyramide Superboost kit (B40935, ThermoFisher Scientific). Goat anti-rat biotinylated antibody (BA-9400-1.5, 2BScientific); Goat anti-rabbit biotinylated antibody (BA-1000-1.5, 2BScientific).

Neutralisation:
Periostin (R&D Systems, AF2955)
Osteopontin (R&D Systems, AF808)
LIF (R&D Systems, AF499)
aCSF1R (BioXCell, BE0213, Clone AFS98)

Validation

All antibodies have been validated by the manufacturer, as indicated in the references provided:

Gapdh, (G9545), Sigma Aldrich: https://www.sigmaaldrich.com/GB/en/product/sigma/g9545
STAT3, (9139), Cell Signaling Technology: https://www.cellsignal.com/products/primary-antibodies/stat3-124h6-mouse-mab/9139
pSTAT3, (9145), Cell Signaling technology: https://www.cellsignal.com/products/primary-antibodies/phospho-stat3-tyr705-d3a7-xp-rabbit-mab/9145
pJAK1, (74219), Cell Signaling Technology: https://www.cellsignal.com/products/primary-antibodies/phospho-jak1-tyr1034-1035-d7n4z-rabbit-mab/74129
JAK1, (3344), Cell Signaling Technology: https://www.cellsignal.com/products/primary-antibodies/jak1-6g4-rabbit-mab/3344
Sortilin, (ab16640), Abcam: https://www.abcam.com/products/primary-antibodies/sortilinnt3-antibody-ab16640.html
alpha tubulin, (T6199), Sigma Aldrich: https://www.sigmaaldrich.com/GB/en/product/sigma/t6199

Osteopontin, (AF808), R&D Systems: https://www.rndsystems.com/products/mouse-osteopontin-opn-antibody_af808
Anti mouse IgG HRP, (7076), Cell Signaling Technology: https://www.cellsignal.com/products/secondary-antibodies/anti-mouse-igg-hrp-linked-antibody/7076
Anti-rabbit IgG HRP, (7074), Cell Signaling Technology: https://www.cellsignal.com/products/secondary-antibodies/anti-rabbit-igg-hrp-linked-antibody/7074
Anti-goat IgG HRP, (ab97120), Abcam: https://www.abcam.com/products/secondary-antibodies/donkey-goat-igg-hl-hrp-preadsorbed-ab97120.html

CD45, (30F-11), Biolegend: https://www.biolegend.com/nl-be/products/purified-anti-mouse-cd45-antibody-102?GroupID=BLG1932
F4/80, (BM8), Biolegend: https://www.biolegend.com/en-gb/products/apc-anti-mouse-f4-80-antibody-4071
Cd11b, (M1/70), Biolegend: https://www.biolegend.com/en-gb/products/pe-anti-mouse-human-cd11b-antibody-349
CD31, (390), Biolegend: https://www.biolegend.com/en-gb/products/apc-anti-mouse-cd31-antibody-118?GroupID=BLG2420
Epcam, (G8.8), Biolegend: https://www.biolegend.com/nl-be/products/apc-anti-mouse-cd326-ep-cam-antibody-4974
CD8a, (53-6.7), Biolegend: https://www.biolegend.com/en-gb/products/percp-cyanine5-5-anti-mouse-cd8a-antibody-4255
IFNg, (XMG1.2), Biolegend: https://www.biolegend.com/en-gb/products/pe-anti-mouse-ifn-gamma-antibody-997?GroupID=GROUP24
Ki67, (ab15580), Abcam: https://www.abcam.com/products/primary-antibodies/ki67-antibody-ab15580.html
aSMA, (ab7187), Abcam: https://www.abcam.com/products/primary-antibodies/alpha-smooth-muscle-actin-antibody-1a4-ab7817.html

Cytokeratin-19, (ab53119), Abcam: https://www.abcam.com/products/primary-antibodies/cytokeratin-19-antibody-ab53119.html
Cytokeratin-19, (ab52625), Abcam: https://www.abcam.com/products/primary-antibodies/cytokeratin-19-antibody-ep1580y-cytoskeleton-marker-ab52625.html
F4/80 (70076), Cell Signaling Technology: https://www.cellsignal.com/products/primary-antibodies/f4-80-d2s9r-xp-rabbit-mab/70076
GFP, (ab6556), Abcam: https://www.abcam.com/products/primary-antibodies/gfp-antibody-ab6556.html
PDGFRb, (ab69506), Abcam: https://www.abcam.com/products/primary-antibodies/pdgfr-beta-antibody-42g12-ab69506.html
CD68, (MO81401-2), Dako: https://www.agilent.com/store/en_US/Prod-M081401-2/M081401-2
Pdgfra, (ab203491), Abcam: https://www.abcam.com/products/primary-antibodies/pdgfr-alpha-antibody-epr22059-270-ab203491.html
aSMA, (ab5694), Abcam: https://www.abcam.com/products/primary-antibodies/alpha-smooth-muscle-actin-antibody-ab5694.html
CD34, (ab8158), Abcam: https://www.abcam.com/products/primary-antibodies/cd34-antibody-mec-147-ab8158.html
CD34, (M082329-2), DAKO: https://www.agilent.com/store/en_US/Prod-M082329-2/M082329-2
CD31 (77699, Cell Signaling Technology: https://www.cellsignal.com/products/primary-antibodies/cd31-pecam-1-d8v9e-xp-rabbit-mab/77699
MCAM, (ab75769), Abcam: https://www.abcam.com/products/primary-antibodies/cd146-antibody-epr3208-ab75769.html
Desmin, (ab32362), Abcam: https://www.abcam.com/products/primary-antibodies/desmin-antibody-y66-cytoskeleton-marker-ab32362.html
YM-1, (60130), Stem Cell Technologies: https://www.stemcell.com/products/anti-ym1-antibody-polyclonal.html
CD8a, (ab22378), Abcam: https://www.abcam.com/products/primary-antibodies/cd8-alpha-antibody-yts1694-ab22378.html
GranzymeB, (AF1865), R&D systems: https://www.rndsystems.com/products/mouse-granzyme-b-antibody_af1865
Anti-mouse IgG AF-488, (ab150105), Abcam: https://www.abcam.com/products/secondary-antibodies/donkey-mouse-igg-hl-alexa-fluor-488-ab150105.html
Anti-rabbit IgG AF-488, (ab150077), Abcam: https://www.abcam.com/products/secondary-antibodies/goat-rabbit-igg-hl-alexa-fluor-488-ab150077.html
Anti-rat IgG AF 594, (ab150160), Abcam: https://www.abcam.com/products/secondary-antibodies/goat-rat-igg-hl-alexa-fluor-594-ab150160.html
Anti-rabbit IgG AF-594, (ab150080), Abcam: https://www.abcam.com/products/secondary-antibodies/goat-rabbit-igg-hl-alexa-fluor-594-ab150080.html
Anti-rat IgG AF-647, (ab150155), Abcam: https://www.abcam.com/products/secondary-antibodies/donkey-rat-igg-hl-alexa-fluor-647-preadsorbed-ab150155.html
Anti-rabbit IgG AF488, (406404), Biolegend: https://www.biolegend.com/en-gb/products/dylight-488-donkey-anti-rabbit-igg-minimal-x-reactivity-5693?GroupID=BLG3874
Anti-rabbit IgG AF594, (406418), Biolegend: https://www.biolegend.com/en-gb/products/alexa-fluor-594-donkey-anti-rabbit-igg-minimal-x-reactivity-9837?GroupID=BLG3472
Anti-rabbit IgG AF647, (406414), Biolegend: https://www.biolegend.com/en-gb/products/alexa-fluor-647-donkey-anti-rabbit-igg-minimal-x-reactivity-9379?GroupID=BLG3472
Superboost Goat Anti-rabbit Poly HRP (B40962), ThermoFisher Scientific: https://www.thermofisher.com/order/catalog/product/B40962
Streptavidin Tyramide Superboost kit (B40935), ThermoFisher Scientific: https://www.thermofisher.com/order/catalog/product/B40935
Goat anti-rat biotinylated antibody, (BA-9400-1.5), 2BScientific: https://vectorlabs.com/products/biotinylated-goat-anti-rat-igg
Goat anti-rabbit biotinylated antibody, (BA-1000-1.5), 2BScientific: https://www.2bscientific.com/Products/VECT/BA-1000-15/Goat-Anti-Rabbit-IgG-Antibody-HL-Biotinylated.
Periostin neutralizing antibody (R&D Systems, AF2955): https://www.rndsystems.com/products/mouse-periostin-osf-2-isoform-2-antibody_af2955
Osteopontin neutralizing antibody (R&D Systems, AF808): https://www.rndsystems.com/products/mouse-osteopontin-opn-antibody_af808
LIF neutralizing antibody (R&D Systems, AF499): https://www.rndsystems.com/products/mouse-lif-antibody_af449
aCSF1R neutralizing antibody (BioXCell, BE0213, Clone AFS98): https://bioxcell.com/invivomab-anti-mouse-csf1r-cd115-be0213

Eukaryotic cell lines

Policy information about cell lines and Sex and Gender in Research

Cell line source(s)	PDAC cells (KPC - FC1199) derived from KPC mice on C57BL/6 background were provided by Dr. David Tuveson at the Cold Spring Harbor Laboratory. LX2 cells were donated by Profesor Jelena Mann, with approval from Professor Scott Friedmann. HEK293T cells were obtained from ATCC.
Authentication	HEK293T and LX2 cells were authenticated by the suppliers by STR profiling. KPC cells were authenticated by genotyping PCR.
Mycoplasma contamination	All cell lines were routinely tested negative for mycoplasma.
Commonly misidentified lines (See ICLAC register)	No commonly misidentified cell lines were used in this study.

Animals and other research organisms

Policy information about studies involving animals; ARRIVE guidelines recommended for reporting animal research, and Sex and Gender in Research

Laboratory animals	C57BL/6 mice were purchased from Charles River Laboratories. Grn -/- mice (B6(Cg)-Grntm1.1Aidi/J) were purchased from the Jackson Laboratory. Pdgfrb-eGFP mice on the C57BL/6 genetic background was kindly gifted by Prof. Neil C. Henderson, Edinburgh. KPC tissue was kindly provided by Prof. Jennifer P. Morton. cKO-STAT3 mice, housed at the CNIO (Madrid), were generated by breeding GFAP-Cre/ERT2 (B6.Cg-Tg(GFAP-Cre/ERT2)505Fmv/J; 012849, Jackson laboratory) with STAT3loxP/loxP mice. All animal experiments with the cKO-STAT3 mice were performed at the CNIO (Madrid) and in accordance with a protocol approved by the CNIO, Instituto de Salud Carlos III and Comunidad de Madrid Institutional Animal Care and Use Committee. Animals of 6-8 weeks old were utilised in studies.<br>Mice were housed under specific-pathogen-free conditions at the Biomedical Science Unit at the University of Liverpool. Mice were housed under 12h dark/light cycle, 20–24°C, and 45-65% relative humidity. Mice were maintained with environmental enrichment, access to standard chow and water ad libitum.
Wild animals	No wild animals were used in this study
Reporting on sex	Reporting on sex differences has not been collected. In all studies, we used both female and male, apart from STAT3i survival study in which only female mice were used. In all experiments, mice were sex, age, and weight matched across groups and we did not observe any significant differences between the outcome of experiments using females compared to males.
Field-collected samples	No field collected samples were used in the study
Ethics oversight	This study complies with all relevant ethical regulations. All animal studies were conducted in accordance with UK Home Office regulations under project license P16F36770. The maximum tumour burden limit of 1.5cm mean diameter was not exceeded in the studies. In all animal studies, the severity was limited to moderate.

Note that full information on the approval of the study protocol must also be provided in the manuscript.

Flow Cytometry

Plots

Confirm that:

- [x] The axis labels state the marker and fluorochrome used (e.g. CD4-FITC).
- [x] The axis scales are clearly visible. Include numbers along axes only for bottom left plot of group (a 'group' is an analysis of identical markers).
- [x] All plots are contour plots with outliers or pseudocolor plots.
- [x] A numerical value for number of cells or percentage (with statistics) is provided.

Methodology

Sample preparation	Single-cell suspensions from murine livers were prepared by mechanical and enzymatic disruption with 1 mg/mL Collagenase P (Roche) in Hanks Balanced Salt Solution (HBSS) at 37oC for 30-40 minutes. Cells were then incubated with 0.05% trypsin at 37oC for 5 minutes. After removal of debris by filtering the cell suspension through a 70um strainer, red blood cells were removed using RBC Lysis Buffer (Biolegend).<br>Liver cell suspensions were then resuspended in MACS buffer (0.5% BSA, 2mM EDTA, PBS). Fc receptors were blocked using anti-mouse CD16/CD32 (BD Biosciences) for 10 minutes on ice. For cell surface staining, cells were then incubated with SYTOX Blue viability marker (Thermo Fisher) and fluorophore-conjugated antibodies (Biolegend, Supplementary Table 12).<br>For the T cell activation assay, following Fc receptor blocking, cells were incubated LIVE/DEAD™ Fixable Aqua Dead Cell Stain Kit (Thermo Fisher) and fluorophore-conjugated CD8 antibody (Biolegend). Cells were then fixed using IC Fixation Buffer and permeabilised using Intracellular Staining Perm Wash Buffer (Biolegend) according to the manufacturer's instructions, followed by staining with fluorophore-conjugated IFNg antibody.

Instrument	FACSCanto II<br>FACSAria III (for cell sorting)
Software	BD FACSDiva software
Cell population abundance	FACS-sorted cell abundance and purity was determined using FACSDiva software
Gating strategy	Single cells were gated based on FSC-A and FSC-H. Live cells were gated based on Sytox or LIVE/DEAD staining. GFP+ MAFs were gated based on a dump channel of CD45-;CD31-;Epcam-; GFP+ cells. Cancer cells were gated based on viable, Epcam+ staining. Immune cells were gated based on viable, CD45+ staining. Cytotoxic T cells were gated based on viable, CD8+ staining.

☒ Tick this box to confirm that a figure exemplifying the gating strategy is provided in the Supplementary Information.
